# Genomic structure of nucleotide diversity among Lyon rat models of metabolic syndrome

**DOI:** 10.1186/1471-2164-15-197

**Published:** 2014-03-14

**Authors:** Man Chun John Ma, Santosh S Atanur, Timothy J Aitman, Anne E Kwitek

**Affiliations:** Department of Pharmacology, University of Iowa, Iowa City, IA USA; Physiological Genomic and Medicine group, MRC Clinical Sciences Centre, Imperial College London, Hammersmith Campus, London, UK; Iowa Institute of Human Genetics, Department of Pharmacology, Internal Medicine, Molecular Physiology & Biophysics, University of Iowa, 3111B MERF, 375 Newton Rd, 52242 Iowa City, IA USA

**Keywords:** Metabolic syndrome, Rat genetic model, Genetic mapping, Genome sequence, Nucleotide diversity, Evolution

## Abstract

**Background:**

The metabolic syndrome (MetS), a complex disorder involving hypertension, obesity, dyslipidemia and insulin resistance, is a major risk factor for heart disease, stroke, and diabetes. The Lyon Hypertensive (LH), Lyon Normotensive (LN) and Lyon Low-pressure (LL) rats are inbred strains simultaneously derived from a common outbred Sprague Dawley colony by selection for high, normal, and low blood pressure, respectively. Further studies found that LH is a MetS susceptible strain, while LN is resistant and LL has an intermediate phenotype. Whole genome sequencing determined that, while the strains are phenotypically divergent, they are nearly 98% similar at the nucleotide level. Using the sequence of the three strains, we applied an approach that harnesses the distribution of Observed Strain Differences (OSD), or nucleotide diversity, to distinguish genomic regions of identity-by-descent (IBD) from those with divergent ancestry between the three strains. This information was then used to fine-map QTL identified in a cross between LH and LN rats in order to identify candidate genes causing the phenotypes.

**Results:**

We identified haplotypes that, in total, contain at least 95% of the identifiable polymorphisms between the Lyon strains that are likely of differing ancestral origin. By intersecting the identified haplotype blocks with Quantitative Trait Loci (QTL) previously identified in a cross between LH and LN strains, the candidate QTL regions have been narrowed by 78%. Because the genome sequence has been determined, we were further able to identify putative functional variants in genes that are candidates for causing the QTL.

**Conclusions:**

Whole genome sequence analysis between the LH, LN, and LL strains identified the haplotype structure of these three strains and identified candidate genes with sequence variants predicted to affect gene function. This approach, merged with additional integrative genetics approaches, will likely lead to novel mechanisms underlying complex disease and provide new drug targets and therapies.

**Electronic supplementary material:**

The online version of this article (doi:10.1186/1471-2164-15-197) contains supplementary material, which is available to authorized users.

## Background

Metabolic Syndrome (MetS) is a constellation of disorders which include obesity, insulin resistance or hyperglycemia, dyslipidemia and hypertension, the combination of which have been found to significantly increase the risk for cardiovascular disorders and type II diabetes [1]. According to data compiled by the National Health and Nutrition Examination Survey in 2009, more than one-third of the U.S. population falls into the criteria for metabolic syndrome [2], making it a major public health issue. Diagnosis of MetS is made with the co-occurrence of any three of the defining features [1]. While the associated features often occur together and have clear genetic contribution, the common pathways or mechanisms linking them in MetS is not well understood.

Identification of the genetic contribution to complex disease is greatly aided by comprehensive studies involving genetic models. The Lyon inbred rat strains were derived in the early 1970s from a single outbred Sprague–Dawley (SD) colony for different blood pressure levels: hypertension (Lyon Hypertensive; LH/Mav), normotension (Lyon Normotensive; LN/Mav) and hypotension (Lyon Low-pressure; LL/Mav) [3]. While LN rats have normal blood pressure, LL rats have late onset hypotension while LH rats are spontaneously hypertensive by 5 weeks of age [4, 5]. Initially established as a model of hypertension, several defining features of the metabolic syndrome (MetS) have also been observed in LH [1, 6]. These include obesity, dyslipidemia with an increase in total triglycerides, total cholesterol, and increased insulin and insulin:glucose ratio, which suggests a susceptibility to insulin resistance [4, 6, 7]. Therefore the LH rat is a MetS susceptible rat. The study of the Lyon strains, having differing genetic susceptibilities to traits defining MetS, can be used to dissect the underlying genetic causes of the defining features of a disorder that carries a significant health burden [8, 9].

We previously identified quantitative trait loci (QTL) for phenotypes defining MetS in an F2 intercross between LH and LN rats, including body weight, blood pressure, plasma lipid levels, and plasma insulin levels [10]. While many of the traits were influenced by QTL on different chromosomes, this study determined that rat chromosome (RNO) 17 contains QTLs for multiple features of MetS (body weight; blood pressure; plasma cholesterol, triglyceride, and insulin levels). While blood pressure and plasma lipid levels were correlated in the F2 cross, body weight was not found to be correlated with either of these traits [6], suggesting the QTL on RNO17 for body weight may have been due to the co-segregation of a passenger locus during selection rather than the pleiotropic effect of a single MetS gene on this chromosome.

Because the inbred strains were derived from a single SD colony, the Lyon strains share high genetic similarity. Phylogenetic studies consistently find the LH, LN, and LL strains in a well-defined cluster of SD-derived inbred rat strains [11–13]. The shared lineage between LH and LN strains also resulted in a paucity of informative polymorphic markers between the strains; therefore, the QTL intervals in our previous mapping study were large, and generating congenic and consomic strains by marker-assisted selection was a challenge. Consomic strains introgressing the more genetically divergent BN chromosomes 13 or 17 succeeded in recapitulating some of the phenotypes – body weight, triglycerides, and blood pressure – that were identified in the QTL analysis [14, 15]. However, the genetic similarity between the Lyon strains presents an opportunity to utilize haplotype mapping to fine-map the loci, if sufficient polymorphic markers could be identified.

In 2007, the STAR Consortium released genotypes for 163 inbred rat strains, including the LH and LN strains, from a 20,238-SNP panel [12]. As was previously determined using microsatellite markers [11], phylogenetic analyses for the rat strains using the 20 K SNP panel indicated a close genetic relationship between the LH and LN strains. Of the 20,238 SNPs in the panel, only 1,739 (8.59%) are polymorphic between LH and LN. Furthermore, the variants clustered into what could be considered putative LD blocks. We assert the genetic determinants for the LH phenotypes reside in LD blocks that differ between the strains, due to artificial selective sweeps from the SD progenitors. Yet, like any SNP genotyping panels, the STAR Consortium panel, determined by an ascertainment panel consisting of SS/Jr, GK/Ox, SHRSP/Bbb, WKY/Bbb and F344/Stm strains [12], is subject to the ascertainment biases observed in SNP panels in general [16] that can impart large effects on many metrics of linkage disequilibrium [17]. Resequencing of the genomes eliminates SNP genotyping biases and allows for more accurate LD analyses; however until recently only a few rat strains had available genome sequence: BN/SsNHsD [18], SHR/OlaIpcv [19], and SD.

We previously determined the single nucleotide polymorphism (SNP) density across the genomes of the SHR and BN strains as a means to visualize the substantial diversity between the two strains [19]. When plotting the genome-wide distribution of SNPs between the strains, we observed a bimodal distribution with one peak in the distribution curve having a low SNP density and the other having a high SNP density [19]. The Observed Strain Differences (OSD), or the density of variants between two strains across a fixed genome sequence window size, represent a *local* measure of polymorphic sites.

Recently we published the genome sequences of 27 different inbred rat strains including the LH, LL, and LN strains [13]. In this study we reported data regarding artificial selective sweeps among the rat strains, and suggest that shared genetic material between strains originating from the same founder population, irrespective of their phenotype, reflects their common ancestry. Considering LH and LN rats were generated through selective breeding from a common origin, we assert the regions with low SNP density are likely regions of shared lineage while the regions with high density would likely to be from different ancestral chromosomes that contain genetic determinants of their phenotypes due to artificial selection from the founder outbred SD rats. As reported here, OSD analysis was performed in the Lyon rat strains in order to fine-map the QTL, particularly on RNO17, and identify candidate genes relating to MetS in the LH rat by comparing sequence variation in this strain to that of the other Lyon strains.

## Results

### Genome-wide Observed Strain Difference (OSD) analyses

For the OSD analyses, six comparisons were performed in two groups. First, each of the three Lyon strains was compared with the BN reference genome (LH/BN; LN/BN; LL/BN). Second, all possible pairwise comparisons between the Lyon strains (LH/LN; LH/LL; LL/LN) was performed to identify regions of the genomes between the strains with ancestrally distinct haplotypes derived from the outbred SD rats.

**Figure 1:**
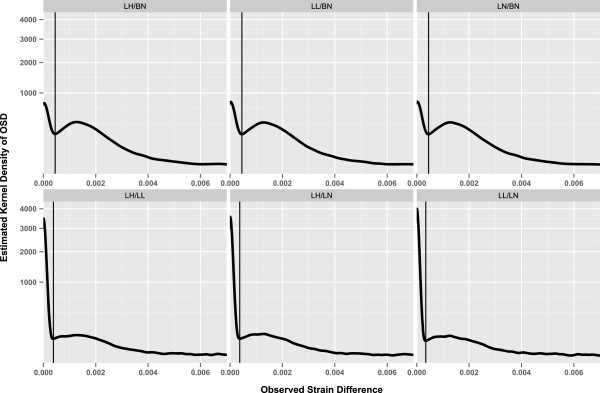
**Distributions of Observed Strain Difference (OSD) over 100Kb windows.** OSD distribution is represented as the curve of kernel density estimates (Y axis) against OSD (X axis). The scale of the Y-axis is square-root transformed. The Polymorphism Enrichment Thresholds (PET) for each comparison is marked with a vertical line.

In all comparisons (Figure 1), the OSD distribution of the 27,199 100Kb-windows spanning the rat genome is bimodal, as was previously reported in the comparison between SHR and BN strains [19]. The first (left) peak in the bimodal distribution contains regions of the genome identical by descent, with OSD values close to zero (i.e. low SNP density). The second (right) peak in the bimodal distribution contains regions of the genome that are ancestrally divergent between the two strains, having high OSD values (i.e. high SNP density). A distinct valley separates the two peaks; we define the OSD value at this valley as the *Polymorphism Enrichment Threshold* (PET). The average PET in the Lyon vs. BN and the pairwise Lyon strain comparisons is 4.5 × 10^−4^ and 3.7 × 10^−4^, respectively (Table 1). Regions with SNP density values higher than the PET represent the windows within ancestral haplotype blocks that differ between the strains.

**Table 1:** **Summary Statistics for OSD analyses for six strain comparisons**

Comparison	PET	# Windows > PET	% Windows > PET	# divergent haplotype blocks	Average block length	SD block length	Average OSD first peak	Average OSD second peak	# SNPs in comparison	# SNPs in haplotype blocks	% SNPs in haplotype blocks
**Lyon vs BN**											
LH/BN	4.50x10^−4^	18,242	67.07	1,431	1,431,996	1,506,679	5.44x10^−5^	1.69x10^−3^	3,127,650	3,078,898	98.44
LL/BN	4.52x10^−4^	17,989	66.14	1,396	1,287,865	1,565,760	5.10x10^−5^	1.69x10^−3^	3,094,251	3,047,301	98.48
LN/BN	4.57x10^−4^	17,982	66.11	1,398	1,285,500	1,493,916	5.05x10^−5^	1.69x10^−3^	3,082,757	3,036,234	98.49
Average	4.53x10^−4^	18,071	66.44	1,408	1,335,120	1,522,118	5.20x10^−5^	1.69x10^−3^	3,101,553	3,054,144	98.47
**Lyon pairwise**											
LH/LN	3.69x10^−4^	4,202	15.45	477	880,419	960,896	5.28x10^−6^	1.50x10^−3^	643,234	630,814	98.07
LH/LL	3.84x10^−4^	4,041	14.86	485	828,584	851,988	5.68x10^−6^	1.56x10^−3^	643,233	630,878	98.08
LL/LN	3.44x10^−4^	3,510	12.90	360	968,953	1,048,667	3.74x10^−6^	1.49x10^−3^	532,429	531,904	99.90
Average	3.66x10^−4^	3,918	14.40	441	892,652	953,850	4.90x10^−6^	1.52x10^−3^	606,299	597,865	98.68

Comparing the SNP densities between the groups of comparisons, distinct differences in the nature of the distribution curves were observed (Figure 1). While all comparisons show a bimodal distribution, the number of windows with low SNP density (and accordingly low OSD values) is approximately 4-fold higher in the Lyon pairwise group than in the Lyon vs BN group. Conversely, the number of windows with high SNP density (high OSD values) is over 3-fold lower in the Lyon pairwise groups compared to the Lyon vs BN groups. This trend is consistent with the fact that the Lyon strains are evolutionarily close to each other but evolutionary distant from the BN strain [12]. It also explains the low amount of polymorphism between the Lyon strains as compared to the Lyon vs BN comparisons (Table 1). The percentage of 100Kb windows with high SNP density increases from an average of 14.40% in Lyon pairwise comparisons to 66.44% in Lyon vs BN comparisons.

In order to determine haplotype blocks between the strains being compared, adjacent windows with SNP density exceeding the PET were concatenated. There were 3-fold more divergent haplotype blocks in the Lyon vs BN comparisons compared to the Lyon pairwise comparisons, with an average of 1,408 in Lyon vs BN groups compared to an average of 441 in Lyon pairwise groups (Table 1). Furthermore, the divergent haplotypes in the Lyon strains comparisons were on average less than 0.9 Mb in length, whereas the Lyon vs BN haplotypes were nearly 50% longer, with an average of over 1.3 Mb. Together, these data are consistent with the breeding history of the Lyon rat strains.

**Figure 2:**
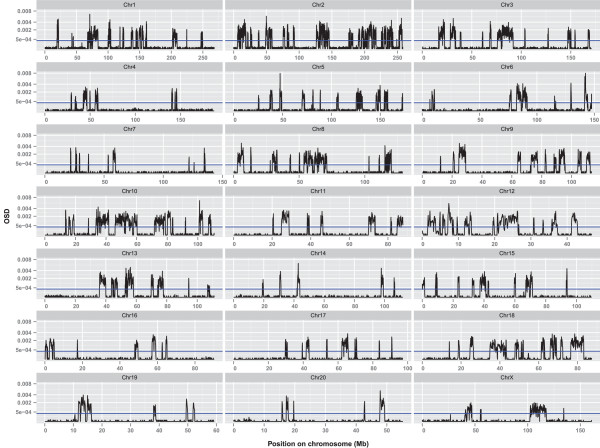
**Patterns of OSD Distribution over 100Kb across the genome in the LH/LN comparison.** The scale of the Y-axis is square-root transformed.

The haplotype blocks were then aligned to the reference BN sequence to determine their distribution in the rat genome. Regardless of the pairwise comparison, the distribution of haplotype blocks across the genome was highly variable. For example, in the LH/LN comparison (Figure 2, Additional file 1: Table S1), approximately 15.5% of the genome contains divergent haplotype blocks. In comparison, nearly 31% of chromosomes 2, 10 and 12 are comprised of divergent haplotype blocks, while only approximately 5% of chromosomes 7, 14 and 20 encompass divergent haplotype blocks. The latter three chromosomes also have long stretches of 50 Mb or more where there is no window exceeding the PET, that is, regions that are shared ancestrally.

**Figure 3:**
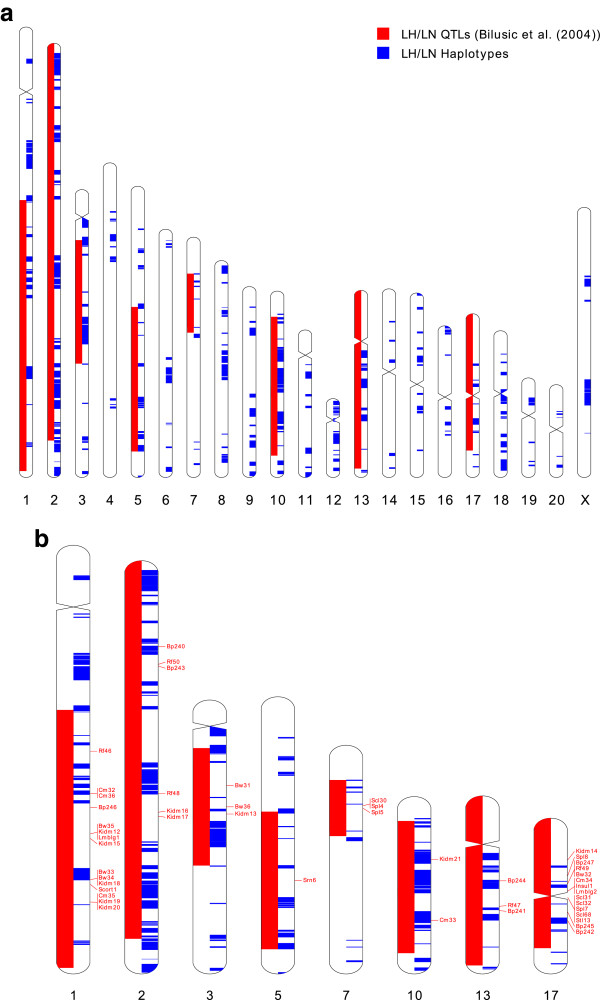
**The overlap between previously reported QTLs and the divergent haplotype blocks between LH and LN. (A):** Genome-wide comparison; **(B):** A focus on the chromosomes 1, 2, 3, 5, 7, 10, 13 and 17 that contains at least one QTL identified by Bilusic et al. In both figures red marks QTL intervals identified by Bilusic et al. and blue marks intervals containing divergent haplotype blocks. Idiograms were drawn using Idiographica [21].

Because of the phenotype-driven selection of the Lyon strains from a common SD ancestor, it is likely that divergent haplotypes arising from artificial selective sweeps will contain variants causing the phenotypic differences between the strains. In order to fine-map QTL intervals for MetS traits previously mapped in a cross between LH and LN rats, we aligned both the haplotype blocks and QTL onto the rat genome and determined where the two overlap [10]. Using the genomic coordinates provided by the Rat Genome Database [20], the QTL intervals cover a total of ~860 Mb bp, or 33% of the entire rat genome (Figure 3a). However, only 21% of these intervals (183 Mb) contain haplotypes differing between LH and LN strains. Therefore, these studies allow for *in silico* fine-mapping of QTL intervals, narrowing them by nearly 80%, and particularly on the chromosomes with relatively few divergent haplotypes such as chromosomes 7 and 17 (Figure 3b, Additional file 1: Table S1).

### Patterns of Haplotype Blocks on RNO17

**Figure 4:**
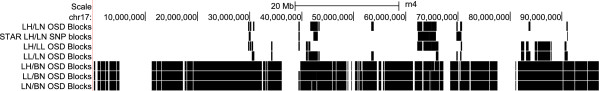
**Divergent haplotype blocks of different comparisons on RNO17.** From top: LH/BN, LL/BN, LN/BN, LH/LL, LH/LN and LL/LN. At the bottom the LH/LN haplotype blocks identified by SNP genotyping was added as reference.

Despite strong evidence that RNO17 has genetic determinants contributing to multiple symptoms of MetS, the paucity of markers polymorphic between LH and LN presents a particular challenge to fine-map the genetic loci on this chromosome. Therefore, here we applied the OSD-based approach to RNO17 to fine-map the genetic loci identified in the cross between LH and LN rats (Figure 4). When comparing the Lyon vs. BN groups to the Lyon pairwise groups, it is clear that the majority of the chromosome is divergent in the Lyon vs. BN comparison with only small haplotype blocks in common, while the vast majority of RNO17 is conserved among the Lyon strains. We identified 14 haplotype blocks on RNO17 that differ between the LH and LN strains (Table 2). The span of these blocks cover 7.5 Mb, or 7.7% of the chromosome, and contain 11,852 of 12,175 SNPs (97.3%) between LH and LN rats on this chromosome identified by resequencing (Table 2, Additional file 1: Table S1). The percentage of RNO17 representing ancestrally different haplotype blocks are half of the genomic average of 15.4%, further demonstrating the similarity between LH and LN strains on this chromosome.

**Table 2:** **Divergent haplotype blocks between LH and LN strains identified by OSD analysis and SNP genotyping data**

	Haplotype blocks OSD analysis			Haplotype blocks STAR genotyping	
Block number	Start (mb)	End (mb)	Length (mb)	Start (mb)	End (mb)
1	29.7	30.0	0.3	29.7	30.2
2	30.1	30.3	0.2		
3	30.7	30.9	0.2	*novel*	
4	39.4	39.6	0.2	38.2	39.8
5	41.7	43.1	1.4	42.2	43.2
6	43.2	43.4	0.2		
7	53.4	53.8	0.4	*novel*	
8	62.2	63.2	1.0	62.3	65.8
9	63.4	64.9	1.5		
10	65	65.9	0.9		
11	69.7	69.9	0.2	69.7	70.8
12	70.4	70.8	0.4		
13	83.6	83.9	0.3	*novel*	
14	90.8	91.1	0.3	90.8	90.9

All genome position coordinates are based on the rn4 assembly.

The LH and LN strains have previously undergone genome-wide SNP genotyping by the STAR consortium [12]. From these genotyping results we deduced a list of putative haplotype blocks on RNO17 and compared them to the OSD-based results (Table 2). The haplotype blocks identified by both approaches are largely similar, with both identifying blocks at 29–30 Mb, 39 Mb, 42–43 Mb, 62–65 Mb, 69–70 Mb and 91 Mb. However, the present approach identified three novel putative haplotype regions at 30.7-30.9 Mb, 53.4-53.8 Mb, and 83.6-83.9 Mb. In addition, while both approaches identified a haplotype block ending at approximately 43.1 Mb, the start site of the block as identified by OSD analysis extends the 5′end by approximately 500 Kb compared to the one identified by SNP genotyping (41.7 vs 42.2 Mb, respectively), making the block about 47% longer. On the other hand, SNP genotyping identified a 1.6 Mb haplotype block spanning 38.2-39.8 Mb, while OSD analysis refined this block to 0.2 Mb (39.4-39.6 Mb), which can largely be attributed by the full map resolution provided by resequencing. Overlaying the haplotypes with the mapped QTL implicate blocks 1–12 as most likely to contain causal genes for the mapped traits.

### Genes and SNVs located in Haplotype Blocks

Using the OSD analysis to identify ancestrally different haplotypes allows us to focus initial efforts identifying causal genes for the QTL in the LH rat. The 477 haplotypes divergent between LH and LN contain 3,687 protein-coding genes; 1,789 of these genes fall within one or more of the previously identified QTLs [10]. The resequencing of the Lyon strains identified 643,234 SNPs and 327,067 indels across the genome in the LH/LN comparison, of which 630,814 and 235,414 are located in the haplotype blocks [13]. Genome-wide, there are 2,391 SNPs and 542 indels in the LH/LN comparison that Variant Effect Predictor (VEP) [22] classified as causing non-synonymous coding, frameshift, splice site changes, and/or stop codon gain/loss. Nearly all of these are located in the haplotype blocks, including 2,083 SNVs and 383 indels in 1,316 genes. Overlaying these SNVs and indels with QTL regions identified 416 genes with putative functional variation between the LH and LN strains.

On chromosome 17, there are 27 protein-coding genes located within the haplotype blocks differing between LH and LN strains (Table 3). All except 2 of these genes fell within one or more of the previously reported QTLs associated with LH phenotypes (Figure 3b) [10]. We have identified 24 SNVs and 7 indels in 15 genes on RNO17 classified as affecting protein sequence, or splice sites, by VEP (Table 3). Each of these variants fell within one of the haplotype blocks differing between LH and LN strains. Of the 31 variants, 18 variants in 11 genes were the minor allele in the LH rat, and were colocalized with MetS QTL. There were three genes (*RGD1563300*, *Prl5a2*, and *Prl4a1*) with LH variants affecting splice sites and three genes (*Prl4a1*, *ENSRNOG00000012418,* and *LOC364753*) with variants that were classified as “probably damaging” or “possibly damaging” by PolyPhen 2 version 2.2.2 [23].

**Table 3:** **Genes and non-synonymous variations in LH vs LN haplotype blocks on RNO17**

Gene name	Gene start (bp)	Gene end (bp)	Description	Nucleotide Substitution	AA Substitution	Variant strain	Classification
**Block 1 (29.7-30 Mb)**							
*Tmem14c*	29,701,923	29,708,044	Transmembrane protein 14C				
*Pak1ip2*	29,710,407	29,721,477	PAK1 interacting protein 1				
*RGD1562963*	29,733,271	29,746,769	Similar to chromosome 6 open reading frame 52	**G29,733,378A**	V36I	LH	Benign
				**G29,741,903A**	R135H		Benign
				**G29,741,915A**	C139Y		Benign
*Gcnt2*	29,767,388	29,872,873	N-acetyllactosaminide beta-1,6-N-acetylglucosaminyl-transferase	C29,872,483 T	A131T	LN	Benign
**Block 2 (30.1-30. Mb)**							
*LOC100362620*	30,267,398	30,267,637	CDC28 protein kinase regulatory subunit 2	G30,267,440A	E15K	LN	Benign
				T30,267,495C	L33P		Benign
				G30,267,526C	W43C		Benign
				G30,267,578 T	E61*		N/A
**Block 5 (41.7-43.1 Mb)**							
*RGD1563300*	42,228,845	42,229,431	Similar to 60S ribosomal protein L29 (P23)	g.42299020_42299027delACTCCGGT		LH	Essential splice site
				g.42299028_42299029insCACAAAGATA	X29fs	LN	Frameshift
*Prl5a2*	42,984,939	42,991,275	Prolactin family 5, subfamily a, member 2	G42,989,474A	P14L	LH	Benign
				A42,986,191 T		LH	Splice site
**Block 6 (43.2-43.4 Mb)**							
*Prl5a1*	43,119,152	43,126,570	Prolactin-5A1				
*Prl4a1*	43,276,214	43,284,152	Prolactin-4A1	**G43,278,266 T**	T141N	LH	Splice site, possibly damaging
**Block 7 (53.4-53.8 Mb)**							
*Stard3nl*	53,402,078	53,436,081	MLN64 N-terminal domain homolog				
*ENSRNOG00000027571*	53,441,303	53,484,317	Uncharacterized protein	G53,441,394A	T161I /T313I*	LH	Benign
				C53,463,467 T	V124I	LH	
				g.53483901_53483997del	73_105del	LN	Frameshift
*ENSRNOG00000012418*	53,496,423	53,528,130	Uncharacterized protein	**G53,527,707 T**	P81T	LH	Possibly damaging
				**T53,527,779A**	T57S	LH	Benign
				**G53,528,005A**	P15S	LH	Probably damaging
				**G53,528,025 T**	A8D	LH	Possibly damaging
*Amph*	53,558,804	53,802,936	Amphiphysin	C53,558,811A	R632L	LH	Benign
				g.53641892_53641893delCT	c.152_153delAG	LN	
*ENSRNOG00000038737*	53,773,036	53,773,733	Uncharacterized protein				
**Block 8 (62.2-63.2 Mb)**							
*Bambi*	62,654,080	62,658,885	BMP and activin membrane-bound inhibitor homolog				
*RGD1564129*	62,684,244	62,686,747	Uncharacterized protein				
*Cul2*	62,701,289	62,741,344	Cullin-2				
*Crem*	62,770,633	62,837,668	cAMP-responsive element modulator				
*Epc1*	63,041,415	63,104,046	Enhancer of polycomb homolog 1	A63,102,600 T	L55H	LH	Benign
**Block 9 (63.4-64.9 Mb)**							
*Rab18*	63,497,924	63,529,227	Ras-related protein Rab-18	A63,528,108 T	S193C	LN	Benign
*Mkx*	63,631,426	63,710,099	Mohawk Homeobox	G63,631,814A	P301L	LH	Benign
*Armc4*	63,931,393	63,955,410	Armadillo repeat containing 4				
*Mpp7*	63,992,387	64,282,554	MAGUK p55 subfamily member 7				
*Wac*	64,531,772	64,587,027	WW domain containing adaptor with coiled-coil	T64,570,567G	C200G	LH	Benign
**Block 10 (65.0-65.9 Mb)**							
*LOC364753*	65,681,793	65,702,382	similar to NSFL1 (p97) cofactor (p47)	**G65,701,876 T**	G80C	LH	Possibly damaging
**Block 13 (83.6-83.9 Mb)**							
*ENSRNOG00000031981*	83,837,499	83,861,361	Uncharacterized protein	C83,860,804 T	P40S	LH	Benign
				g.83861107_83861122delATCCCTGCATCCCTGC	I141fs	LN	Frameshift
				g.83861220_83861227delCCCTGCAT	T178fs	LH	Frameshift
				**g.83837957_83837958insA**		LH	Splice site
**Block 14 (90.8-91.1 Mb)**							
*Plxdc2*	90,572,391	90,982,073	Plexin domain-containing protein 2				

Variants in bold were validated by Sanger sequencing.

To interrogate the SNVs’ possible roles in MetS traits, Fisher’s exact test was performed to test whether the LH allele SNVs listed in Table 3 are significantly enriched among the sequenced rat strains [13] that have one or more symptoms of MetS: obesity, dyslipidemia and hypertension. One variant in *LOC364753* (17:G65,701,876 T) showed significant enrichment at *p* < 0.05; it was found to be enriched (p = 0.01) among the hypertensive LH, SS and SHR strains.

### Variant confirmation

To verify the existence of SNVs within the haplotype blocks on RNO17, we performed Sanger sequencing of 6 amplicons containing 10 of the variants listed in Table 3 (Additional file 2: Table S2). These six amplicons generated a total of 3,848 base pairs of sequence. All 10 variants were validated by Sanger sequencing, with the LH and LN allele identical to genome resequencing results. Furthermore, we were able to verify 20 of the 23 SNVs that were annotated in the genome sequence and identified an additional SNV that was not previously annotated. These results reflect the high quality of the genome sequence of the strains.

## Discussion

In this paper we report a simple technique to distinguish genomic regions of identity-by-descent (IBD) from those with different ancestry using genome resequencing results from a group of rat strains that shares a common origin but were selectively inbred for differing phenotypes. Genetic studies in phenotype-selected inbred rodent strains derived from a common ancestor are a common strategy to map loci for many complex disorders, ranging from anxiety [24, 25] to hypertension [26]. The similar genetic background strains minimizes the heterogeneity outside of the regions phenotypically selected, making identity-by-descent (IBD) mapping a means to eliminate disease-causing regions of the genome. However, their similar genetic backgrounds also present problems to the investigator, as their similarities result in a paucity of polymorphic markers available to attain an acceptable marker resolution for mapping. Using next-generation sequencing (NGS) techniques to resequence the genomes of these strains can resolve this problem as NGS, by definition, samples all bases, and hence should be able to identify all polymorphisms between strains, allowing high-resolution IBD mapping. In the case of the Lyon strains, which share similar SD ancestors, we distinguished ancestral haplotypes that have been fixed in the course of selective inbreeding from the random mutations that were fixed after the division of the strains in order to fine-map QTL for traits defining MetS.

The results presented here confirm previous data regarding the genomes of different laboratory mouse strains, which also observed bimodal distributions of SNP densities in non-overlapping windows across the genome [27, 28]. By resequencing a selection of putative SNPs from each peak, Wade et al. found that SNPs identified in the low SNP density regions are likely to be spurious, while those identified on the high SNP density regions are likely to be validated [27]. Furthermore, by comparing the distribution of nucleotide diversity (π) [29] among synonymous SNPs in cDNA transcripts in laboratory mouse strains, wild-derived mouse strains (control for high diversity), as well as a rat strain from a single founder (control for low diversity), Reuveni and colleagues assert that the bimodal distribution of π in laboratory mice is contributed by two groups of SNPs: intra-subspecific SNPs and inter-subspecific SNPs, represented by the low π and high π peaks respectively [30]. While that paper mainly discusses mouse subspecies, we expect the implication can also be extrapolated to strain differences. In this case, SNPs that were represented by the low- π peak (or the low-OSD peak in this case) are likely to be SNPs that arise after the separation of the strains, while SNPs that were represented by the high- π peak, or high-OSD peak, represents SNPs that originate from the genetic differences between the founder strains.

The observed bimodal distribution of SNP density has previously been reported by Wang et al. in a similar comparison between the *indica* and *japonica* subspecies of rice, using microarray genotyping and a window size of 200 kB [31]. Furthermore, whole-genome resequencing between individual strains within the *indica* subspecies showed similar results as lower-density SNP typing [32].

The evolutionary histories of rice the Lyon strains are different. However, artificial selection from a single origin was put forward by the authors as an explanation for the bimodal distribution of SNP density. Since the Lyon strains were in fact artificially selected from a single origin based on their blood pressure, we consider the authors’ conclusion about the relationship between the distribution of SNP densities and phenotype in rice can be applied to the Lyon rats. Specifically, the non-IBD regions between LH and LN contain genetic determinants for the divergence between LH and LN phenotypes. This approach has also been used among mammals, to identify genomic regions that underlie the domestication of dogs using whole-genome sequence [33].

A caveat to our approach is the assumption that the phenotype differences between the Lyon rat strains are due to phenotype-driven selection of ancestrally different loci. While we cannot formally rule out that random mutation after divergence of the strains does have some phenotypic outcome, given the multigenic nature of the traits, we assert this approach will identify at least a subset of the disease-causing variants. In addition, we cannot confirm the method described in this paper is able to identify all divergent haplotype blocks between two similar strains, particularly in genome regions lacking adequate coverage. However this approach is appropriate to prioritize genetic loci that may contain genetic determinants for the phenotype in question which can be verified in vivo by using consomic and/or congenic strains [34].

In the Lyon pairwise comparisons, no more than 15% of the 100 Kb blocks on the genome have been identified as divergent haplotype blocks, yet these blocks contain more than 97% of all identifiable SNPs in the comparisons (Table 1). Specifically, in the LH/LN comparison, divergent haplotype blocks encompass 420.0 Mb of the rat genome. QTL intervals mapped in a cross between the two strains encompass 827 Mb of the genome. Combining the QTL and haplotype mapping narrowed the loci by nearly 80% to 183 Mb [10], allowing a more refined focus for gene discovery.

As mentioned previously, multiple QTL for MetS traits were mapped to RNO17 in an LH × LN F2 intercross [10]. However, the QTL intervals span nearly the entire chromosome due to the relative low density of the genetic map. The approach reported here allowed for *in silico* fine-mapping of the QTL by narrowing the possible candidate regions and thus reducing the number of candidate genes to 25. Of these, 11 are protein-altering variants in the LH rat, 5 of which are predicted to negatively impact function. Two prolactin genes (*Prl5a2* and *Prl4a1)* have variants predicted to be damaging in the LH rat. Interestingly, low serum prolactin levels have been reported to be associated with MetS in humans, both women and men [35, 36]. Furthermore, plasma prolactin levels were found to be significantly decreased in the GK rat, an inbred model of type 2 diabetes. Interestingly in a cross between GK and BN rats, plasma prolactin levels were linked rat chromosome 17 in male rats [37]. Furthermore, the GK and LH rats share the same haplotypes for these genes, as do BN and LN strains. However, at this locus, the GK allele was actually associated with *higher* plasma prolactin levels. Therefore the impact of the variants in these prolactin genes is unclear.

The remaining three genes with predicted functional variants in the LH rat either had no known function (*RGD1563300* and *Loc364753*) or no previously reported relationship with MetS, such as *ENSRNOG00000012418,* which has sequence similarity with T cell receptor gamma variable genes (*TRGV*).

Other genes in the haplotype blocks may not have non-synonymous variants characterized as ‘benign’ by prediction software, but have been associated with symptoms of MetS in previous research. Haplotype block 1 contains *RGD1562963*, a rat ortholog of human *C6ORF52*. The bovine ortholog of *RGD1562963* falls within bovine QTL223, involving in beef marbling, i.e. the deposition of fat in bovine muscles [38, 39]. This gene contains three non-synonymous SNPs in LH, albeit the prediction software categorized as the variants as being “benign.” *Amphiphysin* (*Amph)* is a gene in haplotype block 7 with a nonsynonymous mutation causing a R632L amino acid change that is categorized as “benign” by PolyPhen2 in the LH rat*.* While loss of function mutations in this gene are known to cause Stiff Person Syndrome [40], a SNP in *Amph* is also associated with sagittal diameter (a measure of central obesity) in the Framingham Heart Study 100 K dataset [41]. LH rats also have a nonsynonymous (C200G) mutation in *Wac*, a gene that may be essential in Golgi biosynthesis [42]. Interestingly, the variant in *Wac* is unique to LH and SS strains and could thus underlie their shared phenotypes of hypertension [13].

Finally, other genes in the haplotypes underlying the MetS QTL on LH chromosome 17, have no identified coding variants, but have notable function related to MetS. Blocks 8 to 10, separated by two 100Kb windows, includes several genes of note. *Bambi* is a protein that modifies TGF-beta signals by acting as a pseudo-receptor [43]. Knocking out *Bambi* in the mouse results in a weight decrease in females [44] and an increase in arterial wall neovascularization [45]. *Cul2* is part of the VHL tumor suppression complex that ubiquitinates HIF1α [46]; the disruption of HIF1α has been found to improve the insulin sensitivity and decrease adiposity in mice [47]. Also, mutations in another member of the cullin family, *CUL3*, have been found to cause some Mendelian forms of hypertension [48]. *Crem* is an inducible CREB repressor whose down-regulation has been shown to contribute to insulin resistance in obese human and mice through the resulting increase in CREB expression [49], and mouse knockout models show protection against cardiopathy and left ventricular dysfunction, especially after exposure to β1-adrenergic agonists [50, 51]. Finally, *Mpp7*, which has been determined to cause at least one case of Maturity-onset diabetes of the young (MODY) [52] and has been associated with left ventricular hypertrophy, BMI and incidence of cardiovascular diseases in the Framingham study [53–55]. Of note, this region falls in the peak of linkage for blood pressure and plasma lipid QTL previously mapped in the LH × LN intercross [10]. While these genes are interesting candidate genes, further studies are required, for example in congenic strains, to establish their roles in MetS.

We could only identify one non-synonymous SNV on the haplotype blocks of RNO17 that has the LH allele overrepresented among strains having MetS symptoms, a variant in *LOC364753* (17:G65,701,876 T) that showed significant enrichment among the strains LH, SS and SHR. Interestingly, this is an LH variant predicted to be ‘possibly damaging.’ However, the *variant* T allele is actually conserved across vertebrates; therefore it is not likely to play a causal role in our phenotypes. Furthermore, many of these variants are rare, which may decrease the power of the Fisher’s exact test. For example, two of the non-synonymous variations in *RGD1562963* are only observed in LH and SR/Jr among the sequenced strains (both derived from SD rats), and the variation causing non-synonymous mutations in *Wac* are only observed in LH and SS rats. While the coselection of genes common to hypertension is obvious in the LH and SS strains in the case of *Wac*, the shared alleles in *RGD1562963* between LH and SR strains in relation to MetS is less obvious. While SR rats are commonly studied as a normotensive model of the salt-sensitive SS/Jr, they actually have elevated body weights compared to SS rats [56, 57]. Therefore, while performing association studies in inbred strains may identify some genes for MetS, the heterogeneity of the phenotypes and their underlying causes complicate gene discovery. In fact, our analyses across multiple inbred rat strains that are models of hypertension, obesity, and dyslipidemia found no genes in common between all disease strains [13]. Furthermore, because the traits defining MetS are multigenic traits in themselves, some risk alleles may be present in ‘normal’ strains but are insufficient to independently influence the phenotype. Therefore it is important to have genetic data from QTL mapping studies or congenic strains to confirm the *in silico* findings.

## Conclusions

We utilized the ancestral history of the selective inbreeding in the Lyon rat stains to identify LD blocks likely to harbor causal genes by analyzing the OSD distribution arising from the genome resequencing and overlaying them with QTL. Using this approach we have been able to identify a group of genes on RNO17 that may contribute to the traits underlying MetS in the LH rat strain.

The resequencing of several inbred rat strains including the Lyon strains provides a remarkable resource for identifying genes causing some of the most common human diseases, such as metabolic syndrome and cardiovascular disease. The sequence is the final component to round out integrative genetic approaches to identify novel MetS genes and we anticipate this resource will result in the identification of many novel mechanisms of and therapies for one of the most common diseases of the 21st century.

## Methods

### Genome resequencing

The genome sequence of the LH, LN, and LL rats was performed previously as described [13]. All animal protocols were reviewed and approved by the IACUC at the University of Iowa. Briefly, DNA was extracted from the spleens of two individuals each from LH (LH/MavRrrcAek), LL (LL/MavRrrcAek) and LN (LN/MavRrrcAek) strains, followed by 100 bp paired-end sequencing of 300–600 bp fragments on an Illumina Hiseq 2000 platform as previously described [13]. Reads were then aligned to the RGSC-3.4 rat reference genome [18] with the Burrows-Wheeler Aligner version 0.5.8c [58]. The Genome Analysis Toolkit version 1.0.6001 [59, 60] was then used to discover and genotype genomic variations. Variants were called from reads mapped with mapping quality greater than 10 and bases with base quality greater or equal to 17, with the variant scores thereafter recalibrated and filtered using GATK’s GMM model [60]. Sequencing gaps were identified as regions of zero coverage from the output of BEDTools’s [61] genomecov function (Additional file 3: Table S3).

### OSD analysis

Observed Strain Differences (OSD) of non-overlapping 100-kb windows across the genome were calculated as previously described [19]. OSD was defined as the number of identified SNVs between the strains (where each strain’s genotype is homozygous) within a 100 kb window divided by the number of nucleotides in that window that have a definitive sequence call in all the strains being compared. For all comparisons, only positions that have passed quality control and are homozygous across all strains within the comparison were used in OSD calculation.

The distribution density of OSD amongst all windows across the genome were first smoothed by binned kernel density estimate [62] as implemented by the R [63] package KernSmooth with default parameters. This means estimating the kernel density on 401 equally spaced points with a Gaussian kernel and with bandwidth estimated by Wand and Jones’ oversmoothed kernel selector. From the kernel smoothing results, a *Polymorphism Enrichment Threshold* (PET) was determined, defined as the OSD value which is located in the local OSD minimum after the first local OSD maximum. Putative blocks of LD were generated by identifying and merging contiguous 100 kb windows with OSD values greater or equal to PET. These blocks represent haplotypes that differ between the strains being compared.

### Downstream analyses

Genes that are located within the haplotype blocks were identified using Ensembl version 69 [64] gene annotations as provided by Ensembl BioMart [65] in the Rn4 assembly. The effects of the identified SNVs and indels were predicted by Ensembl’s Variant Effect Predictor (VEP) [22] based on Ensembl version 69 data and using Ensembl consequence terms. For the purpose of this paper, non-synonymous variations are defined as variations containing the term NON_SYNONYMOUS_CODING as the predicted consequence. Similarly, splice sites variants are defined by terms SPLICE_SITE and ESSENTIAL_SPLICE_SITE, frameshift variants by the term FRAMESHIFT_CODING, and stop-gained variation by the term STOP_GAINED from the VEP output. PolyPhen version 2.2.2 [23] was used to predict the effects of SNPs identified as nonsynonymous by VEP, based on UniProt 2012_09 data [66].

The genotypes of SNVs located within the LH/LN haplotype blocks on RNO17 that have been annotated to cause non-synonymous mutations or splice-site mutations among the rat strains sequenced by Atanur et al. [13] were obtained from Variant Visualizer within the Rat Genome Database [20]. Potential enrichment of the LH allele among the obese strains (LH, SBH, SS, SHR, LL and LEW), dyslipidemic strains (LH, SS and SHR) and hypertensive strains (LH, FHH, MHS, SBH, SHR, SHRSP and SS) against the other strains were statistically tested using two-tailed Fisher’s exact test. In this analysis all substrains of BN were not used as they were considered identical to the reference sequence. In addition, the strain BBDP/Rhw was also not used out of concern that the Type I diabetes phenotype may be confounding.

SNP genotyping-based haplotype blocks between LH and LN strains in RNO17 were identified by visual inspection for contiguous regions of polymorphism from the STAR SNP genotype panel [12]; the haplotype blocks are defined to be the regions between the flanking monomorphic SNPs surrounding the regions of polymorphic SNPs.

### Variant confirmation

Seven non-synonymous variants on the haplotype blocks on RNO17 listed on Table 3 were confirmed by Sanger sequencing (Additional file 2: Table S2). Primers for these amplicons were designed by Primer-BLAST [67] using the region 1 Kb upstream and downstream of the variation as template, with M13 sequence (5′-TGT AAA ACG ACG GCC AGT-3′) tagged at the 5′ ends of the forward primer sequences and another M13 sequence (5′-GTG TGG AAT TGT GAG CGG -3′) tagged to the 5′ ends of the reverse primer sequences. Sequence was based on the rn4 assembly, with the exception of one amplicon. Because the flanking region downstream of the variation at 17:43,278,266 contained a large stretch of gaps in the rn4 assembly, the sequencing primer set for this variation was designed using coordinating location in the rn5 assembly (17:40,575,021).

PCR amplification was performed and products were purified by gel electrophoresis and then sequenced bidirectionally using the M13 primers listed above using ABI 3730xl sequencer with BigDye version 3.1 chemistry (Life Technologies). Sequence traces were aligned to the genome using SeqMan version 9.1.0 (DNASTAR Inc., Madison, WI, USA). SNVs were validated if both strains had sequence passing QC and base-calling was unambiguous.

### Data deposition and availability of supporting data

All sequence data was deposited in the EBI Sequence Read Archive with accession number ERP002160 (http://www.ebi.ac.uk/ena/data/view/ERP002160) as reported previously [13]. Sequence variants are available at the Rat Genome Database (RGD; http://rgd.mcw.edu/).

## Electronic supplementary material

Additional file 1: Table S1: Haplotype blocks identified between LH. and LN strains. (DOCX 16 KB)

Additional file 2: Table S2: Sanger sequence validation of selected variants. (DOCX 16 KB)

Additional file 3: Table S3: Sequence coverage in haplotype blocks. (DOCX 15 KB)

## References

[1] Alberti KGMM, Eckel RH, Grundy SM, Zimmet PZ, Cleeman JI, Donato KA, Fruchart J-C, James WPT, Loria CM, Smith SC (2009). Harmonizing the Metabolic Syndrome. Circulation.

[2] Ervin RB (2009). Prevalence of metabolic syndrome among adults 20 years of age and over, by sex, age, race and ethnicity, and body mass index: United States, 2003–2006. Natl Health Stat Report.

[3] Dupont J, Dupont JC, Froment A, Milon H, Vincent M (1973). Selection of three strains of rats with spontaneously different levels of blood pressure. Biomedicine.

[4] Sassolas A, Vincent M, Benzoni D, Sassard J (1981). Plasma Lipids in Genetically Hypertensive Rats of the Lyon Strain. J Cardiovasc Pharmacol.

[5] Su DF, Cerutti C, Barres C, Vincent M, Sassard J (1986). Blood pressure and baroreflex sensitivity in conscious hypertensive rats of Lyon strain. Am J Physiol.

[6] Vincent M, Boussairi EH, Cartier R, Lo M, Sassolas A, Cerutti C, Barres C, Gustin MP, Cuisinaud G, Samani NJ, Lathrop GM, Sassard J (1993). High blood pressure and metabolic disorders are associated in the Lyon hypertensive rat. J Hypertens.

[7] Vincent M, Cartier R, Privat P, Benzoni D, Samani NJ, Sassard J (1996). Major cardiovascular risk factors in Lyon hypertensive rats. A correlation analysis in a segregating population. J Hypertens.

[8] Ogden CL, Carroll MD, Curtin LR, McDowell MA, Tabak CJ, Flegal KM (2006). Prevalence of overweight and obesity in the United States, 1999–2004. Jama.

[9] Thom T, Haase N, Rosamond W, Howard VJ, Rumsfeld J, Manolio T, Zheng ZJ, Flegal K, O'Donnell C, Kittner S, Lloyd-Jones D, Goff DC, Hong Y, Adams R, Friday G, Furie K, Gorelick P, Kissela B, Marler J, Meigs J, Roger V, Sidney S, Sorlie P, Steinberger J, Wasserthiel-Smoller S, Wilson M, Wolf P (2006). Heart disease and stroke statistics–2006 update: a report from the American Heart Association Statistics Committee and Stroke Statistics Subcommittee. Circulation.

[10] Bilusic M, Bataillard A, Tschannen MR, Gao L, Barreto NE, Vincent M, Wang T, Jacob HJ, Sassard J, Kwitek AE (2004). Mapping the genetic determinants of hypertension, metabolic diseases, and related phenotypes in the lyon hypertensive rat. Hypertension.

[11] Thomas MA, Chen C-F, Jensen-Seaman MI, Tonellato PJ, Twigger SN (2003). Phylogenetics of rat inbred strains. Mamm Genome.

[12] Saar K, Beck A, Bihoreau MT, Birney E, Brocklebank D, Chen Y, Cuppen E, Demonchy S, Dopazo J, Flicek P, Foglio M, Fujiyama A, Gut IG, Gauguier D, Guigo R, Guryev V, Heinig M, Hummel O, Jahn N, Klages S, Kren V, Kube M, Kuhl H, Kuramoto T, Kuroki Y, Lechner D, Lee YA, Lopez-Bigas N, Lathrop GM, Mashimo T (2008). SNP and haplotype mapping for genetic analysis in the rat. Nat Genet.

[13] Atanur SS, Diaz AG, Maratou K, Sarkis A, Rotival M, Game L, Tschannen MR, Kaisaki PJ, Otto GW, Ma MC, Keane TM, Hummel O, Saar K, Chen W, Guryev V, Gopalakrishnan K, Garrett MR, Joe B, Citterio L, Bianchi G, McBride M, Dominiczak A, Adams DJ, Serikawa T, Flicek P, Cuppen E, Hubner N, Petretto E, Gauguier D, Kwitek A (2013). Genome Sequencing Reveals Loci under Artificial Selection that Underlie Disease Phenotypes in the Laboratory Rat. Cell.

[14] Gilibert S, Kwitek AE, Hubner N, Tschannen M, Jacob HJ, Sassard J, Bataillard A (2008). Effects of chromosome 17 on features of the metabolic syndrome in the Lyon hypertensive rat. Physiol Genomics.

[15] Gilibert S, Bataillard A, Nussberger J, Sassard J, Kwitek AE (2009). Implication of chromosome 13 on hypertension and associated disorders in Lyon hypertensive rats. J Hypertens.

[16] Albrechtsen A, Nielsen FC, Nielsen R (2010). Ascertainment biases in SNP chips affect measures of population divergence. Mol Biol Evol.

[17] Nielsen R, Signorovitch J (2003). Correcting for ascertainment biases when analyzing SNP data: applications to the estimation of linkage disequilibrium. Theor Popul Biol.

[18] Gibbs RA, Weinstock GM, Metzker ML, Muzny DM, Sodergren EJ, Scherer S, Scott G, Steffen D, Worley KC, Burch PE, Okwuonu G, Hines S, Lewis L, DeRamo C, Delgado O, Dugan-Rocha S, Miner G, Morgan M, Hawes A, Gill R, Celera, Holt RA, Adams MD, Amanatides PG, Baden-Tillson H, Barnstead M, Chin S, Evans CA, Ferriera S, Fosler C (2004). Genome sequence of the Brown Norway rat yields insights into mammalian evolution. Nature.

[19] Atanur SS, Birol I, Guryev V, Hirst M, Hummel O, Morrissey C, Behmoaras J, Fernandez-Suarez XM, Johnson MD, McLaren WM, Patone G, Petretto E, Plessy C, Rockland KS, Rockland C, Saar K, Zhao Y, Carninci P, Flicek P, Kurtz T, Cuppen E, Pravenec M, Hubner N, Jones SJ, Birney E, Aitman TJ (2010). The genome sequence of the spontaneously hypertensive rat: Analysis and functional significance. Genome Res.

[20] Laulederkind SJ, Hayman GT, Wang SJ, Smith JR, Lowry TF, Nigam R, Petri V, de Pons J, Dwinell MR, Shimoyama M, Munzenmaier DH, Worthey EA, Jacob HJ (2013). The Rat Genome Database 2013–data, tools and users. Brief Bioinform.

[21] Kin T, Ono Y (2007). Idiographica: a general-purpose web application to build idiograms on-demand for human, mouse and rat. Bioinformatics (Oxford, England).

[22] McLaren W, Pritchard B, Rios D, Chen Y, Flicek P, Cunningham F (2010). Deriving the consequences of genomic variants with the Ensembl API and SNP Effect Predictor. Bioinformatics (Oxford, England).

[23] Adzhubei IA, Schmidt S, Peshkin L, Ramensky VE, Gerasimova A, Bork P, Kondrashov AS, Sunyaev SR (2010). A method and server for predicting damaging missense mutations. Nature methods.

[24] Ugarte A, Eguibar JR, Cortes Mdel C, Leon-Chavez BA, Melo AI (2011). Comparative analysis of maternal care in the high-yawning (HY) and low-yawning (LY) sublines from Sprague–Dawley rats. Dev Psychobiol.

[25] Yen YC, Mauch CP, Dahlhoff M, Micale V, Bunck M, Sartori SB, Singewald N, Landgraf R, Wotjak CT (2012). Increased levels of conditioned fear and avoidance behavior coincide with changes in phosphorylation of the protein kinase B (AKT) within the amygdala in a mouse model of extremes in trait anxiety. Neurobiol Learn Mem.

[26] Bell R, Herring SM, Gokul N, Monita M, Grove ML, Boerwinkle E, Doris PA (2011). High-resolution identity by descent mapping uncovers the genetic basis for blood pressure differences between spontaneously hypertensive rat lines. Circ Cardiovasc Genet.

[27] Wade CM, Kulbokas EJ, Kirby AW, Zody MC, Mullikin JC, Lander ES, Lindblad-Toh K, Daly MJ (2002). The mosaic structure of variation in the laboratory mouse genome. Nature.

[28] Adams DJ, Dermitzakis ET, Cox T, Smith J, Davies R, Banerjee R, Bonfield J, Mullikin JC, Chung YJ, Rogers J, Bradley A (2005). Complex haplotypes, copy number polymorphisms and coding variation in two recently divergent mouse strains. Nat Genet.

[29] Watterson GA (1975). On the number of segregating sites in genetical models without recombination. Theor Popul Biol.

[30] Reuveni E, Birney E, Gross CT (2010). The consequence of natural selection on genetic variation in the mouse. Genomics.

[31] Wang L, Hao L, Li X, Hu S, Ge S, Yu J (2009). SNP deserts of Asian cultivated rice: genomic regions under domestication. J Evol Biol.

[32] Subbaiyan GK, Waters DL, Katiyar SK, Sadananda AR, Vaddadi S, Henry RJ (2012). Genome-wide DNA polymorphisms in elite indica rice inbreds discovered by whole-genome sequencing. Plant Biotechnol J.

[33] Axelsson E, Ratnakumar A, Arendt ML, Maqbool K, Webster MT, Perloski M, Liberg O, Arnemo JM, Hedhammar A, Lindblad-Toh K (2013). The genomic signature of dog domestication reveals adaptation to a starch-rich diet. Nature.

[34] Kwitek-Black AE, Jacob HJ (2001). The use of designer rats in the genetic dissection of hypertension. Curr Hypertens Rep.

[35] Corona G, Rastrelli G, Boddi V, Monami M, Melani C, Balzi D, Sforza A, Forti G, Mannucci E, Maggi M (2011). Prolactin levels independently predict major cardiovascular events in patients with erectile dysfunction. Int J Androl.

[36] Balbach L, Wallaschofski H, Volzke H, Nauck M, Dorr M, Haring R (2013). Serum prolactin concentrations as risk factor of metabolic syndrome or type 2 diabetes?. BMC Endocr Disord.

[37] Finlay C, Argoud K, Wilder SP, Ouali F, Ktorza A, Kaisaki PJ, Gauguier D (2010). Chromosomal mapping of pancreatic islet morphological features and regulatory hormones in the spontaneously diabetic (Type 2) Goto-Kakizaki rat. Mammalian genome : official journal of the International Mammalian Genome Society.

[38] Eberlein A, Kalbe C, Goldammer T, Brunner RM, Kuehn C, Weikard R (2011). Annotation of novel transcripts putatively relevant for bovine fat metabolism. Mol Biol Rep.

[39] Casas E, Shackelford SD, Keele JW, Koohmaraie M, Smith TP, Stone RT (2003). Detection of quantitative trait loci for growth and carcass composition in cattle. J Anim Sci.

[40] De Camilli P, Thomas A, Cofiell R, Folli F, Lichte B, Piccolo G, Meinck HM, Austoni M, Fassetta G, Bottazzo G, Bates D, Cartlidge N, Solimena M, Kilimann MW (1993). The synaptic vesicle-associated protein amphiphysin is the 128-kD autoantigen of Stiff-Man syndrome with breast cancer. J Exp Med.

[41] Fox CS, Heard-Costa N, Cupples LA, Dupuis J, Vasan RS, Atwood LD (2007). Genome-wide association to body mass index and waist circumference: the Framingham Heart Study 100K project. BMC Med Genet.

[42] Totsukawa G, Kaneko Y, Uchiyama K, Toh H, Tamura K, Kondo H (2011). VCIP135 deubiquitinase and its binding protein, WAC, in p97ATPase-mediated membrane fusion. Embo J.

[43] Onichtchouk D, Chen YG, Dosch R, Gawantka V, Delius H, Massague J, Niehrs C (1999). Silencing of TGF-beta signalling by the pseudoreceptor BAMBI. Nature.

[44] Chen J, Bush JO, Ovitt CE, Lan Y, Jiang R (2007). The TGF-beta pseudoreceptor gene Bambi is dispensable for mouse embryonic development and postnatal survival. Genesis.

[45] Guillot N, Kollins D, Badimon JJ, Schlondorff D, Hutter R (2013). Accelerated reendothelialization, increased neovascularization and erythrocyte extravasation after arterial injury in BAMBI−/− mice. PloS one.

[46] Kamura T, Sato S, Iwai K, Czyzyk-Krzeska M, Conaway RC, Conaway JW (2000). Activation of HIF1alpha ubiquitination by a reconstituted von Hippel-Lindau (VHL) tumor suppressor complex. Proc Natl Acad Sci USA.

[47] Jiang C, Qu A, Matsubara T, Chanturiya T, Jou W, Gavrilova O, Shah YM, Gonzalez FJ (2011). Disruption of hypoxia-inducible factor 1 in adipocytes improves insulin sensitivity and decreases adiposity in high-fat diet-fed mice. Diabetes.

[48] Boyden LM, Choi M, Choate KA, Nelson-Williams CJ, Farhi A, Toka HR, Tikhonova IR, Bjornson R, Mane SM, Colussi G, Lebel M, Gordon RD, Semmekrot BA, Poujol A, Valimaki MJ, De Ferrari ME, Sanjad SA, Gutkin M, Karet FE, Tucci JR, Stockigt JR, Keppler-Noreuil KM, Porter CC, Anand SK, Whiteford ML, Davis ID, Dewar SB, Bettinelli A, Fadrowski JJ, Belsha CW (2012). Mutations in kelch-like 3 and cullin 3 cause hypertension and electrolyte abnormalities. Nature.

[49] Favre D, Le Gouill E, Fahmi D, Verdumo C, Chinetti-Gbaguidi G, Staels B, Caiazzo R, Pattou F, Le KA, Tappy L, Regazzi R, Giusti V, Vollenweider P, Waeber G, Abderrahmani A (2011). Impaired expression of the inducible cAMP early repressor accounts for sustained adipose CREB activity in obesity. Diabetes.

[50] Muller FU, Lewin G, Matus M, Neumann J, Riemann B, Wistuba J, Schutz G, Schmitz W (2003). Impaired cardiac contraction and relaxation and decreased expression of sarcoplasmic Ca2+−ATPase in mice lacking the CREM gene. FASEB journal : official publication of the Federation of American Societies for Experimental Biology.

[51] Lewin G, Matus M, Basu A, Frebel K, Rohsbach SP, Safronenko A, Seidl MD, Stumpel F, Buchwalow I, Konig S, Engelhardt S, Lohse MJ, Schmitz W, Muller FU (2009). Critical role of transcription factor cyclic AMP response element modulator in beta1-adrenoceptor-mediated cardiac dysfunction. Circulation.

[52] Bhoj EJ, Romeo S, Baroni MG, Bartov G, Schultz RA, Zinn AR (2009). MODY-like diabetes associated with an apparently balanced translocation: possible involvement of MPP7 gene and cell polarity in the pathogenesis of diabetes. Mole Cytogenet.

[53] Larson MG, Atwood LD, Benjamin EJ, Cupples LA, D'Agostino RB, Fox CS, Govindaraju DR, Guo CY, Heard-Costa NL, Hwang SJ, Murabito JM, Newton-Cheh C, O'Donnell CJ, Seshadri S, Vasan RS, Wang TJ, Wolf PA, Levy D (2007). Framingham Heart Study 100K project: genome-wide associations for cardiovascular disease outcomes. BMC Med Genet.

[54] Meigs JB, Manning AK, Fox CS, Florez JC, Liu C, Cupples LA, Dupuis J (2007). Genome-wide association with diabetes-related traits in the Framingham Heart Study. BMC Med Genet.

[55] Vasan RS, Larson MG, Aragam J, Wang TJ, Mitchell GF, Kathiresan S, Newton-Cheh C, Vita JA, Keyes MJ, O'Donnell CJ, Levy D, Benjamin EJ (2007). Genome-wide association of echocardiographic dimensions, brachial artery endothelial function and treadmill exercise responses in the Framingham Heart Study. BMC Med Genet.

[56] Ferrell F, Lanou A, Gray SD (1986). Salt level in weaning diet affects saline preference and fluid intake in Dahl rats. Hypertension.

[57] Nishikimi T, Mori Y, Kobayashi N, Tadokoro K, Wang X, Akimoto K, Yoshihara F, Kangawa K, Matsuoka H (2002). Renoprotective effect of chronic adrenomedullin infusion in Dahl salt-sensitive rats. Hypertension.

[58] Li H, Durbin R (2009). Fast and accurate short read alignment with Burrows-Wheeler transform. Bioinformatics (Oxford, England).

[59] McKenna A, Hanna M, Banks E, Sivachenko A, Cibulskis K, Kernytsky A, Garimella K, Altshuler D, Gabriel S, Daly M, DePristo MA (2010). The Genome Analysis Toolkit: a MapReduce framework for analyzing next-generation DNA sequencing data. Genome Res.

[60] DePristo MA, Banks E, Poplin R, Garimella KV, Maguire JR, Hartl C, Philippakis AA, del Angel G, Rivas MA, Hanna M, McKenna A, Fennell TJ, Kernytsky AM, Sivachenko AY, Cibulskis K, Gabriel SB, Altshuler D, Daly MJ (2011). A framework for variation discovery and genotyping using next-generation DNA sequencing data. Nat Genet.

[61] Quinlan AR, Hall IM (2010). BEDTools: a flexible suite of utilities for comparing genomic features. Bioinformatics (Oxford, England).

[62] Wand MP, Jones MC (1995). Kernel Smoothing.

[63] R Development Core Team (2011). R: A Language and Environment for Statistical Computing.

[64] Flicek P, Amode MR, Barrell D, Beal K, Brent S, Carvalho-Silva D, Clapham P, Coates G, Fairley S, Fitzgerald S, Gil L, Gordon L, Hendrix M, Hourlier T, Johnson N, Kahari AK, Keefe D, Keenan S, Kinsella R, Komorowska M, Koscielny G, Kulesha E, Larsson P, Longden I, McLaren W, Muffato M, Overduin B, Pignatelli M, Pritchard B, Riat HS (2012). Ensembl 2012. Nucleic Acids Res.

[65] Kinsella RJ, Kähäri A, Haider S, Zamora J, Proctor G, Spudich G, Almeida-King J, Staines D, Derwent P, Kerhornou A, Kersey P, Flicek P (2011). Ensembl BioMarts: a hub for data retrieval across taxonomic space. Database.

[66] The UniProt Consortium (2012). Reorganizing the protein space at the Universal Protein Resource (UniProt). Nucleic Acids Res.

[67] Ye J, Coulouris G, Zaretskaya I, Cutcutache I, Rozen S, Madden TL (2012). Primer-BLAST: a tool to design target-specific primers for polymerase chain reaction. BMC Bioinformatics.

