# Supplementary material for: Genomic structure of nucleotide diversity among Lyon rat models of metabolic syndrome
Source: BMC Genomics. 2014 Mar 14;15(1):197. doi: 10.1186/1471-2164-15-197 (PMC4003853; doi:10.1186/1471-2164-15-197)
Supplement: Supplementary file 1 — Additional file 1: Table S1: Haplotype blocks identified between LH. and LN strains. (DOCX 16 KB) [file 12864_2013_7035_MOESM1_ESM.docx]

| **Chromosome** | **Total Length of Haplotype Blocks (bp)** | **Chromosome length (bp)** | **% Chromosome in Haplotype** | **# SNPs on Chromosome** | **# SNPs within Haplotype Blocks** | **% SNPs in Haplotype blocks** |
| --- | --- | --- | --- | --- | --- | --- |
| 1 | 39,000,000 | 267,910,886 | 14.56 | 55,885 | 54,263 | 97.10 |
| 2 | 80,000,000 | 258,207,540 | 30.98 | 129,306 | 127,670 | 98.73 |
| 3 | 34,100,000 | 171,063,335 | 19.93 | 52,302 | 51,840 | 99.12 |
| 4 | 10,400,000 | 187,126,005 | 5.56 | 15,493 | 14,878 | 96.03 |
| 5 | 20,900,000 | 173,096,209 | 12.07 | 29,228 | 28,549 | 97.68 |
| 6 | 14,500,000 | 147,636,619 | 9.82 | 25,808 | 25,164 | 97.50 |
| 7 | 5,600,000 | 143,002,779 | 3.92 | 10,055 | 9,745 | 96.92 |
| 8 | 27,000,000 | 129,041,809 | 20.92 | 40,012 | 39,297 | 98.21 |
| 9 | 21,900,000 | 113,440,463 | 19.31 | 37,167 | 36,716 | 98.79 |
| 10 | 36,400,000 | 110,718,848 | 32.88 | 53,302 | 52,673 | 98.82 |
| 11 | 12,659,784 | 87,759,784 | 14.43 | 19,815 | 19,381 | 97.81 |
| 12 | 14,800,000 | 46,782,294 | 31.64 | 22,858 | 22,364 | 97.84 |
| 13 | 17,900,000 | 111,154,910 | 16.10 | 28,665 | 28,216 | 98.43 |
| 14 | 5,000,000 | 112,194,335 | 4.46 | 9,071 | 8,804 | 97.06 |
| 15 | 13,200,000 | 109,758,846 | 12.03 | 21,840 | 21,350 | 97.76 |
| 16 | 7,900,000 | 90,238,779 | 8.75 | 12,763 | 12,454 | 97.58 |
| 17 | 7,500,000 | 97,296,363 | 7.71 | 12,175 | 11,852 | 97.35 |
| 18 | 24,000,000 | 87,265,094 | 27.50 | 35,028 | 34,479 | 98.43 |
| 19 | 5,500,000 | 59,218,465 | 9.29 | 9,034 | 8,800 | 97.41 |
| 20 | 2,800,000 | 55,268,282 | 5.07 | 5,632 | 5,498 | 97.62 |
| X | 18,900,000 | 160,699,376 | 11.76 | 17,794 | 16,821 | 94.53 |
| Total | 419,959,784 | 2,718,881,021 | 15.45 | 643,233 | 630,814 | 98.07 |

Table S1. Haplotype blocks identified between LH and LN strains.
