# Supplementary material for: Genomic structure of nucleotide diversity among Lyon rat models of metabolic syndrome
Source: BMC Genomics. 2014 Mar 14;15(1):197. doi: 10.1186/1471-2164-15-197 (PMC4003853; doi:10.1186/1471-2164-15-197)
Supplement: Supplementary file 2 — Additional file 2: Table S2: Sanger sequence validation of selected variants. (DOCX 16 KB) [file 12864_2013_7035_MOESM2_ESM.docx]

| **Position on RNO17 (bp)** | **Variant** | **Primer Set** | **Forward Primer** | **Reverse Primer** | **Amplicon start** | **Amplicon End** | **Sequence post Q.C.** | **Additional SNVs confirmed** | **Additional SNVs not confirmed** |
| --- | --- | --- | --- | --- | --- | --- | --- | --- | --- |
| 29733378 | G>A | 1 | 5'-M13F-GAGGTTCTGGCAGCTGGAAG-3' | 5'-M13R-TCATTTGCCTGCCCACTTCT-3' | 29732879 | 29733704 | 649 | 23753165 | 29733150 29733530 29733613 |
| 29741903 | G>A | 2 | 5'-M13F-TGACAGTTTGAATGGCCCGT-3' | 5'-M13R-CGCCAACCCTTGAGCTCATA-3' | 29741504 | 29742376 | 788 | 29741633  29741679  29741708  29741754  29741903  29742040  29742102 | 29741608 |
| 29741915 | G>A |  |  |  |  |  |  |  |  |
| 43278266 | G>T | 3 | 5'-M13F-ACAGAGAAAGTCAGCAAGTTGT-3 | 5'-M13R-AGGAAGAAATGACAGTTGCACA-3 | 43278167 | 43278638 | 380 | 42986413 |  |
| 53527707 | G>T | 4 | 5'-M13F-AGGAAACAGTGCCAAGCCAA-3 | 5'-M13R-GACTGTACTCATGCTGCCCC-3 | 53527390 | 53528156 | 694 | 53527779  53528005  53528005  53528025  53528033 | 53527765 |
| 65701876 | G>T | 5 | 5'-M13F-AATGTCTAGAGGCACAGCTCC-3 | 5'-M13R-CGCTGATCCTGTGACTGTGG-3 | 65701399 | 65702210 | 625 |  |  |
| 83837957-83837958 | insA | 6 | 5'-M13F-GCCAAGCGCCTTTATCCAAC-3 | 5'-M13R-CAACGTCAGACTGGGGCTAC-3 | 83837463 | 83838245 | 712 | 83837973  83838050  83838125  83838187  83838191 |  |

Table S2. Sanger sequence validation of selected variants
