# Supplementary material for: Genomic structure of nucleotide diversity among Lyon rat models of metabolic syndrome
Source: BMC Genomics. 2014 Mar 14;15(1):197. doi: 10.1186/1471-2164-15-197 (PMC4003853; doi:10.1186/1471-2164-15-197)
Supplement: Supplementary file 3 — Additional file 3: Table S3: Sequence coverage in haplotype blocks. (DOCX 15 KB) [file 12864_2013_7035_MOESM3_ESM.docx]

| **Block Number** | **Average LH Coverage (X)** | **Length of gaps in region in LH (bp)** | **Length of LH gaps not attributable to gaps in assembly (bp)** | **Average LN coverage (X)** | **Length of gaps in region in LN (bp)** | **Length of LN gaps not attributable to gaps in assembly (bp)** |
| --- | --- | --- | --- | --- | --- | --- |
| 1 | 22.49 | 8,738 | 1,260 | 20.72 | 23,944 | 2,626 |
| 2 | 21.07 | 5,558 | 2,534 | 21.7 | 5,581 | 2,546 |
| 3 | 22.61 | 11,726 | 1,171 | *23.86* | *5,663* | 1,134 |
| 4 | 26.49 | 2,879 | 923 | 26.26 | 2,898 | 954 |
| 5 | 20.56 | 29,195 | 13,349 | 20.94 | 28,783 | 12,844 |
| 6 | 17.08 | 6,981 | 1,334 | 19.37 | 6,806 | 1,142 |
| 7 | 60.77 | 4,296 | 2,065 | *21.82* | *13,966* | 11,848 |
| 8 | 22.18 | 36,445 | 5,920 | 21.8 | 35,885 | 5,395 |
| 9 | 20.26 | 101,273 | 34,491 | 20.97 | 91,492 | 24,696 |
| 10 | 19.84 | 29,457 | 6,786 | 21.01 | 27,497 | 4,796 |
| 11 | 23.21 | 1,061 | 539 | 23.09 | 830 | 311 |
| 12 | 21.59 | 3,355 | 1,313 | 21.63 | 3,864 | 1,836 |
| 13 | 24.61 | 26,001 | 819 | *23.05* | *25,981* | 798 |
| 14 | 22.08 | 5,828 | 2,121 | 23.08 | 5,471 | 1,657 |

Table S3. Sequence coverage in haplotype blocks
